# Predictive value of ^18^F-fluorodeoxyglucose accumulation in visceral fat activity to detect colorectal cancer metastases (prospective observational cohort study)

**DOI:** 10.12688/f1000research.122814.1

**Published:** 2022-10-10

**Authors:** Amil Suleimanov, Aigul Saduakassova, Denis Vinnikov, Vadim Pokrovsky, Saltanat Mamyrbekova, Anara Daniyarova, Lyaila Kozhabek

**Affiliations:** 1 Faculty of Medicine and Health Care, Al-Farabi Kazakh National University, Almaty, Almaty, 050040, Kazakhstan; 2 Nuclear Medicine Department of the Diagnostic Center, Medical Centre Hospital of President’s Affairs Administration of the Republic of Kazakhstan, Nur-Sultan, Nur-Sultan, 010000, Kazakhstan; 3 Department of biochemistry, Peoples’ Friendship University of Russia (RUDN University), Moscow, Moscow, 117198, Russian Federation; 4 Laboratory of combined therapy of tumors, N.N. Blokhin National Medical Research Center of Oncology, Moscow, Moscow, 115478, Russian Federation

**Keywords:** 18F-FDG, PET/CT, Colorectal cancer, Predictive value

## Abstract

**Background: **To evaluate functional visceral adipose tissue (VAT) activity assessed by ^18^F-fluorodeoxyglucose ( ^18^F-FDG) positron emission tomography/computed tomography (PET/CT) as a predictive factor of metastases in colorectal cancer (CRC) patients.

**Methods:** We reviewed study protocols and PET/CT data of 534 CRC patients; 474 patients were subsequently excluded for various reasons. The remaining 60 patients with histologically confirmed adenocarcinoma were then prospectively assessed and were exposed to ^18^F-FDG PET/CT after a surgical treatment and chemoradiotherapy. Age, histology, stage, and tumor grade data were recorded. Functional VAT activity was verified with maximum standardized uptake value (SUV _max_) using ^18^F-FDG PET/CT and tested as a predictive factor of later metastases in eight subdomains of abdominal regions (RE – epigastric region, RLH – left hypochondriac region, RRL – right lumbar region, RU – umbilical region, RLL – left lumbar region, RRI – right inguinal region, RP – hypogastric (pubic) region, RLI – left inguinal region) and pelvic cavity (P) in the adjusted regression models. In addition, we studied the best areas under the curve (AUC) for SUV _max_ with the corresponding sensitivity (Se) and specificity (Sp).

**Results:** In both adjusted for age regression models and receiver operating characteristic (ROC) curve analysis, ^18^F-FDG accumulation in RLH (cut-off SUV _max_ 0.74; Se 75%; Sp 61%; AUC 0.668; p=0.049), RU (cut-off SUV _max_ 0.78; Se 69%; Sp 61%; AUC 0.679; p=0.035), RRL (cut-off SUV _max_ 1.05; Se 69%; Sp 77%; AUC 0.682; p=0.032) and RRI (cut-off SUV _max_ 0.85; Se 63%; Sp 61%; AUC 0.672; p=0.043) could predict later metastases in CRC patients, as opposed to age, sex, primary tumor location, tumor grade and histology.

**Conclusions:** Functional VAT activity was importantly related to later metastases in CRC patients and can be used as their predictive factor.

## Introduction

Colorectal cancer (CRC) is one of the main causes of high worldwide oncological mortality, and its metastasis to the lymph nodes (LN) is an important prognostic factor. ^1^ Globally, CRC is the third most commonly diagnosed cancer, with an estimated 1.9 million (10%) new cases and 935,173 (9.4%) deaths. ^2^ Furthermore, CRC is the second leading cause of cancer mortalities in 2020, according to the WHO GLOBOCAN database. ^3^ In South-Central Asia, 102,987 (63%) new cases of CRC and 59,206 (36%) mortality incidences were registered in 2020. ^2^^,^^4^

Positron-emission tomography/computed tomography (PET/CT) is a hybrid diagnostic method that shows the value of metabolic processes of the tissue at the molecular level in the tomographic mode. The advantage of PET/CT is to visualize viable tumor tissue and assess its biological activity by the degree of radiopharmaceutical agent accumulation in tissues and can be used to measure the hypermetabolic focus of visceral adipose tissue (VAT) activity. ^18^F-Fluorodeoxyglucose ( ^18^F-FDG) is now extensively used to assess functional VAT activity during PET/CT; therefore, it can identify accumulation loci and further detect metastases. ^5^

Although the predictive role of ^18^F-FDG PET/CT in detecting metastases has been poorly studied, the studies on the reported prognostic value for various cancer locations have yielded inconsistent findings. ^6^^–^^11^ Thus, VAT has been shown to increase the CRC risk, but the relationship between VAT and the predictive outcome in CRC is ambivalent. VAT is closely associated with dysregulated visceral fat activity increasing adipokines related to systemic inflammation, and can play a role in oncogenesis and metastatic lesion. ^1^^,^^12^ The increased inflammatory condition of VAT activity might influence the status of LN in CRC patients. ^13^^–^^17^

Byung Wook Choi *et al.* were among the few to retrospectively show the predictive value of metabolic parameters on ^18^F-FDG PET/CT in classical rectal adenocarcinoma. ^12^ Another study by Sung Hoon Kim *et al.* retrospectively showed the prognostic factor of ^18^F-FDG PET/CT for LN metastasis in rectal cancer, ^18^ whereas Kisoo Pahk *et al.* retrospectively showed the predictive value of functional VAT activity measured by preoperative ^18^F-FDG PET/CT for regional LN or distant metastasis in CRC patients. ^1^

Given that the findings of these studies have been inconsistent in showing the exact maximum standardized uptake value (SUV _max_) readings indicative of a higher risk of metastases, more data is needed to verify whether PET/CT can assist in early metastases identification in CRC patients. Therefore, the objective of this study was to quantitatively define functional VAT activity via ^18^F-FDG PET/CT in patients with CRC and its predictive potential for early LN metastases detection.

## Methods

### Ethical considerations

Approval was obtained from Local Bioethics Commission of the Medical Centre Hospital of President’s Affairs Administration of the Republic of Kazakhstan (approval #17/2020 on 24 January 2020) and Local Ethical Commission of the Al-Farabi Kazakh National University (approval #102 IRB – A102 on 28 May 2020). All patients routinely provided written informed consent for all medical tests and examinations and written informed consent was obtained for participation in the current study. We minimized selection bias by enrolling all patients for whom data were available in the database and of sufficient quality. When calculating the sample size, we allowed the maximum standard deviation. The significance level (α) was 0.05, and the study power was 80%, with a confidence probability of 95% (t=1.96). This study follows the TREND guidelines. ^19^

### Study venue and patients

We enrolled 534 patients with CRC, among which 60 patients had no metastases, 175 patients had metastases, 98 patients had a postoperative relapse with high metabolic activity, and 201 patients had a primary cancer disease progression. Patients who had metastases, postoperative relapse with high metabolic activity and primary cancer disease progression were excluded from the study. In total, we have prospectively evaluated 60 patients with a histologically confirmed diagnosis of adenocarcinoma who underwent ^18^F-FDG PET/CT in the Nuclear Medicine Unit of the Diagnostic Center of the Medical Centre Hospital of President’s Affairs Administration of the Republic of Kazakhstan (Nur-Sultan) during the period time between November 2015 and June 2021.

The study included 60 patients (age 39–81; median 60 (interquartile range (IQR) 55–68) years; 46 women) after a surgical treatment and courses of Folfiri and Folfox chemoradiotherapy according to the regimen. During the initial screening for eligibility, patients with histologically unverified colon cancer or with metastases confirmed at the baseline examination were excluded from the study. We also excluded patients with concurrent cancers. Tumor, lymph nodes, and metastasis (TNM) staging system along with American Joint Committee on Cancer (AJCC) stages of recruited patients are shown in Table 1. As Table 1 presents, there were no patients with AJCC stage IV, whereas adenocarcinoma was identified in 100% of patients. Of note, patients were classified into AJCC stages at their baseline examination, after which they were subjected to treatment and then underwent baseline PET/CT. By the time enrolled patients underwent baseline PET/CT, they had completed their treatment, had no signs of cancer or metastases, and this baseline PET/CT was considered as day 0 of the research.

**Table 1.** Overall baseline patient characteristics.

PTL	Sex	Age (Me)	TNM stage			AJCC stage (n)	Histology (Adenocarcinoma) (n)
	(Female/Male) (n)		T (n)	N (n)	M (n)		
Colon	17/3	60	T _1_ – 1 T _2_ – 2 T _3_ – 4 T _4_ – 13	N _0_ – 9 N _1_ – 8 N _x_ – 3	M _0_ – 20	I – 2 II – 8 III – 10	I – 11 II – 1 III – 1 IV – 7
Sigmoid	16/3	59	T _1_ – 0 T _2_ – 2 T _3_ – 10 T _4_ – 7	N _0_ – 3 N _1_ – 8 N _x_ – 8	M _0_ – 19	I – 0 II – 8 III – 11	I – 8 II – 0 III – 3 IV – 8
Rectum	13/8	61	T _1_ – 0 T _2_ – 5 T _3_ – 14 T _4_ – 2	N _0_ – 9 N _1_ – 5 N _x_ – 7	M _0_ – 21	I – 0 II – 15 III – 6	I – 12 II – 0 III – 4 IV – 5

*Note:* PTL – Primary tumor location; TNM - Tumor, lymph nodes and metastasis staging system; AJCC – American Joint Committee on Cancer; Histology (Adenocarcinoma): I – Well-differentiated; II – Poorly differentiated; III – Low differentiated; IV – Moderate differentiated.

Patients underwent ^18^F-FDG PET/CT at the initial enrollment and then again at a follow-up medical examination scheduled six months or more (median 12, IQR 6–40) after the baseline examination. All images were reconstructed using dedicated workstations and software (image evaluation system Wizard). Patients’ data were anonymized and de-identified before studies.

### ^18^F-FDG PET/CT study protocol and image analysis

^18^F-FDG was produced at the Republican Diagnostic Center (Nur-Sultan, Kazakhstan) and was used on the day of the study due to the ultra-short shelf life (109 minutes). The whole-body ^18^F-FDG PET/CT images were completed using PET/CT scanner (Biograph TruePoint PET·CT, Siemens Medical Solutions USA Inc., USA) and carried out in conformity with the accepted clinical protocol of ^18^F-FDG PET/CT examination. ^20^ Prior to PET/CT procedure and the corresponding ^18^F-FDG injection, patients fasted for at least 6 hours, and the glucose serum level in all patients was <11 mmol/l. The average activity dose of the injected ^18^F-FDG was 252.55 MBk, ranging from 132.5 to 465.3 MBk. The average effective radiation dose was 8.75 mSv, with a range from 6.8 to 17.1 mSv. CT scans were obtained following PET emission scanning. PET/CT study protocol included a topogram, a low dose CT to eliminate signal attenuation and anatomical correlation, and the collection of PET data. Duration of PET data collection depended on the patient’s height and weight, but usually completed within 25–40 minutes. Once PET data were obtained, CT and PET images were reconstructed and stored in the transaxial, coronal, and sagittal slices.

Image analysis was performed using the extended Siemens workspace (Biograph TruePoint PET·CT operating manual) in a region of interest (ROI). We calculated the standardized uptake value accumulation (SUV) in VAT automatically with the software using the formula:

$$\mathrm{SUV}=\left[\mathrm{ROI}\;\left(\mathrm{MBq}/g\right)\right]/\left[\text{injected dose}\;\left(\mathrm{MBq}\right)\right]/\left[\text{total body weight}\;\left(g\right)\right]$$

VAT areas were identified by using predefined Hounsfield units (HU), ranging from [-70] to [-110] from background CT images. To measure the VAT activity, ROI (1.00 mm for each measured point) was divided into regions according to the topographic structure, including eight subdomains of abdominal regions (RE – epigastric region, RLH – left hypochondriac region, RRL – right lumbar region, RU – umbilical region, RLL – left lumbar region, RRI – right inguinal region, RP – hypogastric (pubic) region, RLI – left inguinal region) and pelvic cavity (P). They were located on three consecutive sections of the abdominal cavity to exclude the kidneys’ extra physiological absorption of ^18^F-FDG. We measured SUV _max_ in the axial plane for each area, and the average SUV _max_ of each area was calculated separately. All images were reconstructed in transaxial, sagittal and coronal multiplanar planes and read visually. With these functional parameters, the analysis was carried out by the status of metastatic LN lesions.

### Data analysis and interpretation

The primary end-point of this analysis was SUV _max_ of selected nine locations at baseline and follow-up. Image analysis was performed by determining the maximum standardized uptake value (SUV _max_) VAT accumulation in each abdominal and pelvic cavity point. Each measured point was 1.00 mm and varied depending on the volume of VAT of the measured area. VAT areas were identified from background CT images, and SUV _max_ was defined on PET images, including a hypermetabolic focus on ^18^F-FDG PET/CT. We report SUV _max_ values for nine locations of the VAT, whereas the SUV _max_ at baseline and follow-up was a mean of several loci for each area with a 1-mm shift.

We first tested all variables for normality using the Kolmogorov-Smirnov test. Quantitative variables following the normal distribution pattern are presented as means (M) with the corresponding standard deviation (SD); alternatively, we reported medians with the corresponding IQR. SUV _max_ values for different locations and at different time periods (baseline or follow-up) were then compared with nonparametric tests, such as the Mann-Whitney U-test or Wilcoxon test. Because, in total, we selected nine locations to report SUV _max_ values, we tested SUV _max_ values for each location in the univariate analyses with regard to sex, primary tumor location, and other variables, using either Mann-Whitney U-test (for two groups) or Kruskall-Wallis test (for three or more groups). We also used a similar approach to compare groups depending on metastases status, including positive (pLM) patients in whom metastases were detected at a follow-up visit and negative (nLM) who showed no metastases. In such an analysis, we compared baseline SUV _max_ as a predictor. In addition, we tested age and sex as predictors of showing pLM at follow-up. Locations with significant differences between groups with regard to SUV _max_ and other tested predictors (age, sex) showing significant associations with LM status, were then tested in a logistic regression analysis, first crude, and then adjusted for other significant predictors, where we report the odds ratios (OR) of developing metastases at follow-up with the corresponding 95% confidence intervals (CI).

Finally, we applied receiver operating characteristic (ROC) curve analysis to assess the diagnostic performance of quantitative variables when predicting a categorical outcome. The optimal cut-off value of the quantitative variable was estimated using the Youden’s J statistic. All statistical analyses were performed using StatTech v. 2.7.1 (StatTech LLC, Russia).

## Results

There were more women in the studied group (n=46). The most prevalent primary tumor location (PTL) was the rectum (n=21), n=20 patients had the PTL in the colon, including n=6 as ascending, n=6 as descending and n=8 as transverse, whereas n=19 patients had tumor in the sigmoid, as presented in Table 2. With regard to tumor AJCC classification, most patients were classified as stage II (n=31) and III (n=27), with no patients having stage IV. At the baseline examination, the overall mean SUV _max_ was 0.80, with a significant difference in a nine-group comparison (p=0.016), whereas the highest accumulation level was found in RP (0.89) and the lowest in RLI (0.68). Sex affected the SUV _max_ in RLH (p=0.043) and RLL (p=0.048) locations, yielding higher readings in women compared to men. We also found differences in baseline SUV _max_ for colon, sigmoid and rectum in RRL (p=0.006), RU (p=0.016) and RLL (p=0.004), but not for a histological grade, TNM or AJCC stage ( Table 2). ^21^

**Table 2.** Baseline patients’ SUV _max._

Variable	n (%)	SUV _max_								
		RE	RLH	RRL	RU	RLL	RRI	RP	RLI	P
Sex										
Female	46 (76.7)	0.83	0.81	0.88	0.85	0.90	0.79	0.91	0.74	0.89
Male	14 (23.3)	0.66	0.61	0.76	0.63	0.70	0.60	0.68	0.54	0.70
Primary tumor location										
Colon	20 (33.3)	0.93	0.79	1.08	1.07	0.93	0.92	0.96	0.74	0.89
Rectum	21 (35.0)	0.76	0.69	0.86	0.80	0.93	0.79	0.86	0.81	1.02
Sigmoid	19 (31.7)	0.67	0.61	0.74	0.62	0.78	0.58	0.75	0.59	0.67
T stage										
T _1_	1 (1.7)	0.92	1.18	0.82	1.31	0.67	1.07	1.05	0.75	1.03
T _2_	9 (15.0)	0.77	0.93	0.86	1.07	0.96	0.91	0.92	0.90	1.11
T _3_	28 (46.7)	0.75	0.63	0.82	0.62	0.79	0.67	0.83	0.65	0.73
T _4_	22 (36.7)	0.88	0.72	0.89	0.89	0.87	0.72	0.91	0.71	0.84
N stage										
N _0_	21 (35.0)	0.84	0.76	0.82	0.92	0.87	0.77	0.92	0.75	0.89
N _1_	21 (35.0)	0.75	0.66	0.89	0.76	0.79	0.78	0.87	0.59	0.89
N _x_	18 (30.0)	0.76	0.77	0.86	0.80	0.95	0.63	0.84	0.77	0.76
M stage										
M _0_	60 (100.0)	0.77	0.71	0.86	0.79	0.86	0.73	0.89	0.68	0.87
AJCC stage										
I	2 (3.3)	0.83	0.91	0.93	1.37	1.10	0.99	1.29	1.18	1.11
II	31 (51.7)	0.77	0.68	0.82	0.70	0.86	0.69	0.85	0.70	0.76
III	27 (45.0)	0.76	0.81	0.89	0.80	0.91	0.78	0.91	0.66	0.89
Histology (Adenocarcinoma)										
I	31 (51.7)	0.77	0.62	0.86	0.70	0.81	0.73	0.86	0.66	0.89
II	1 (1.7)	0.74	0.65	1.03	1.44	1.53	0.91	1.53	1.61	1.18
III	8 (13.3)	0.80	0.77	0.78	0.92	0.92	0.68	0.87	0.61	0.78
IV	20 (33.3)	0.80	0.83	0.84	0.96	0.83	0.72	1.00	0.76	0.80

*Note:* SUV _max_ - Maximum standardized uptake value; AJCC – American Joint Committee on Cancer; RE – Epigastric Region; RLH – Left Hypochondriac Region; RRL – Right Lumbar Region; RU – Umbilical Region; RLL – Left Lumbar Region; RRI – Right Inguinal Region; RP – Hypogastric (Pubic) Region; RLI – Left Inguinal Region; P – Pelvic Cavity.

At the follow-up examination of the 60 patients recruited initially, metastases developed in 16 (27%) patients, and these were classified as positive lymphatic metastasis (pLM), whereas the remaining 44 (73%) patients were classified as negative lymphatic metastasis (nLM). Such metastases location included LN of the neck, mediastinum, chest, peritoneum, retroperitoneum, and pelvis. We tested whether baseline SUV _max_ was different in those who developed metastases compared to those who did not. We found that such differences were statistically significant but not for all locations, only for RRL (1.29 vs. 0.82, p=0.032) and RU (1.00 vs. 0.74, p=0.041) ( Table 3), indicative of some predictive potential of SUV _max_ in these two locations for metastasis at follow-up.

**Table 3.** SUV _max_ change overall and two subgroups.

Location	Overall (n = 60)			nLM (n = 44)			pLM (n = 16)			p for B nLM vs pLM
	B	F	p	B	F	p	B	F	p	
RE	0.77	0.98	0.07	0.75	0.98	0.77	0.92	0.99	0.64	0.18
RLH	0.71	0.92	0.07	0.66	0.82	0.06	0.96	0.99	0.75	0.05
RRL	0.86	1.02	0.10	0.82	0.91	0.09	1.29	1.35	0.72	0.03
RU	0.80	0.96	0.07	0.74	0.87	0.08	1.00	1.01	0.49	0.04
RLL	0.87	1.01	0.08	0.86	0.98	0.12	0.95	1.13	0.42	0.47
RRI	0.74	0.77	0.32	0.73	0.75	0.39	0.90	0.95	0.66	0.16
RP	0.89	1.02	0.16	0.89	0.98	0.44	0.89	1.19	0.10	0.83
RLI	0.68	0.82	0.07	0.66	0.78	0.18	0.72	0.86	0.28	0.71
P	0.88	0.97	0.08	0.75	0.95	0.13	0.9	1.04	0.45	0.12
p-value	0.02	0.01		0.04	0.03		0.43	0.15		

*Note:* SUV _max_ - Maximum standardized uptake value; B – Baseline; F – Follow-up; pLM – positive Lymphatic Metastasis; nLM – negative Lymphatic Metastasis; RE – Epigastric Region; RLH – Left Hypochondriac Region; RRL – Right Lumbar Region; RU – Umbilical Region; RLL – Left Lumbar Region; RRI – Right Inguinal Region; RP – Hypogastric (Pubic) Region; RLI – Left Inguinal Region; P – Pelvic Cavity.

The median SUV _max_ of all locations increased from 0.8 at baseline to 0.94 at follow-up (p<0.001). We did not find a statistically significant SUV _max_ increase when considered separately in out of nine locations ( Table 3), mostly because the sample size of each location was only 1/9 of the overall sample. We found the trend of SUV _max_ increase overall when stratified nLM to pLM, but it was insignificant. In addition, follow-up SUV _max_ for colon nLM equaled 0.93, with no difference compared to pLM (1.12; p=0.72). Similarly, we failed to confirm statistically significant differences of SUV _max_ when comparing nLM (0.93) with pLM (0.98) for sigmoid (p=0.62) and rectum (0.92 for nLM and 1.05 for pLM) (p=0.68).

In the univariate analysis, age and sex were not associated with metastases at follow-up (median age in nLM 59 vs. 63 years in pLM, p=0.12). We then tested whether baseline SUV _max_ of the selected two locations found to be significantly associated with metastases at follow-up, including RRL and RU, could predict metastases in the unadjusted and adjusted for age regression models. In the model adjusted for age, the OR for positive metastases at follow-up for RRL was non-significant and equaled 2.88 (95% CI 0.79; 10.70), and this model accounted for only 8% variability, whereas the OR for RU in a similar model adjusted for age was significant and equaled 5.42 (95% CI 1.20; 24.50), with an even greater R ^2^ (0.13).

**Figure 1.**
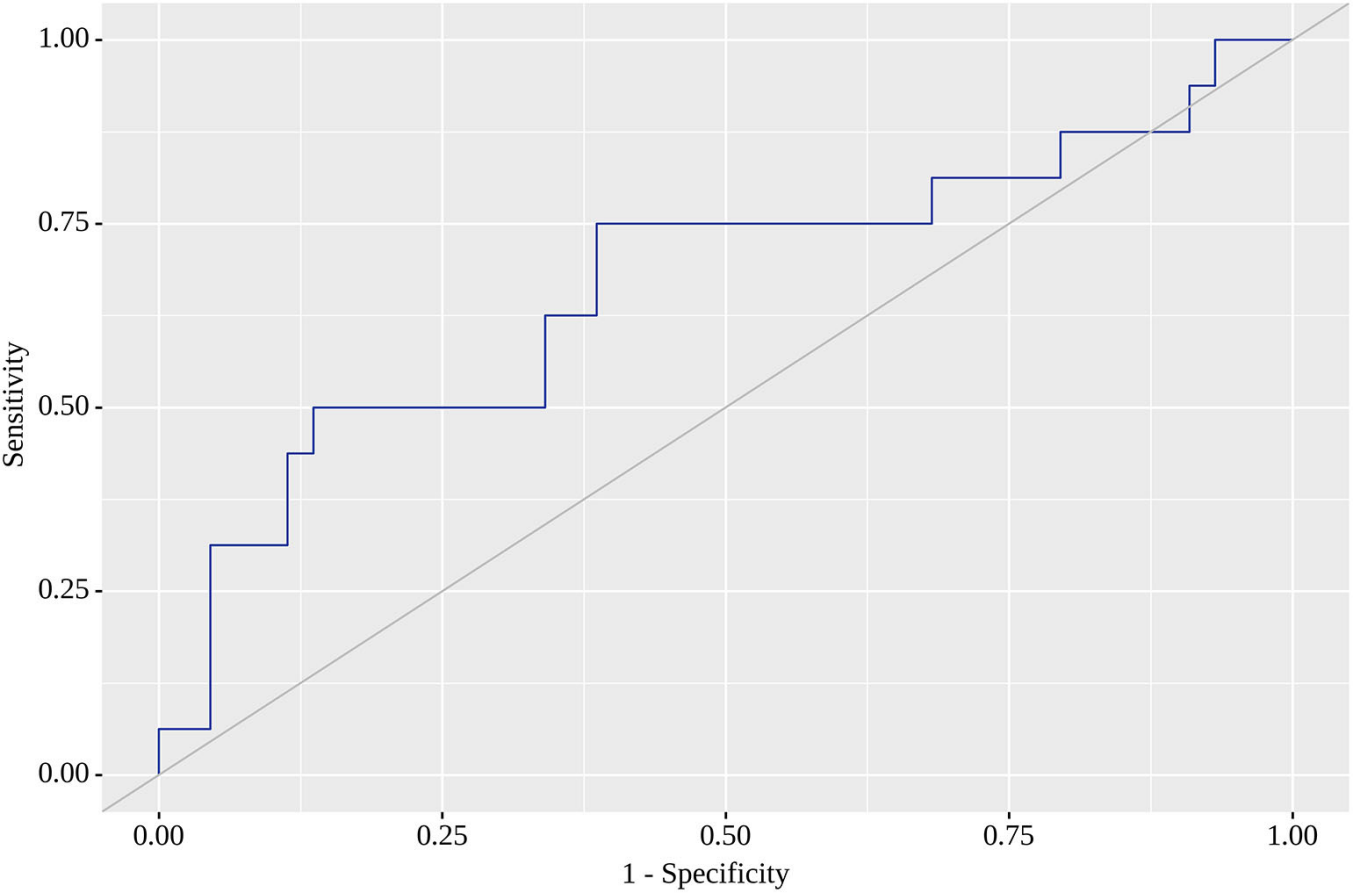
Receiver operating characteristic (ROC) curve showing areas under the curve (AUC) for positive outcome in left hypochondriac region (RLH).

**Figure 2.**
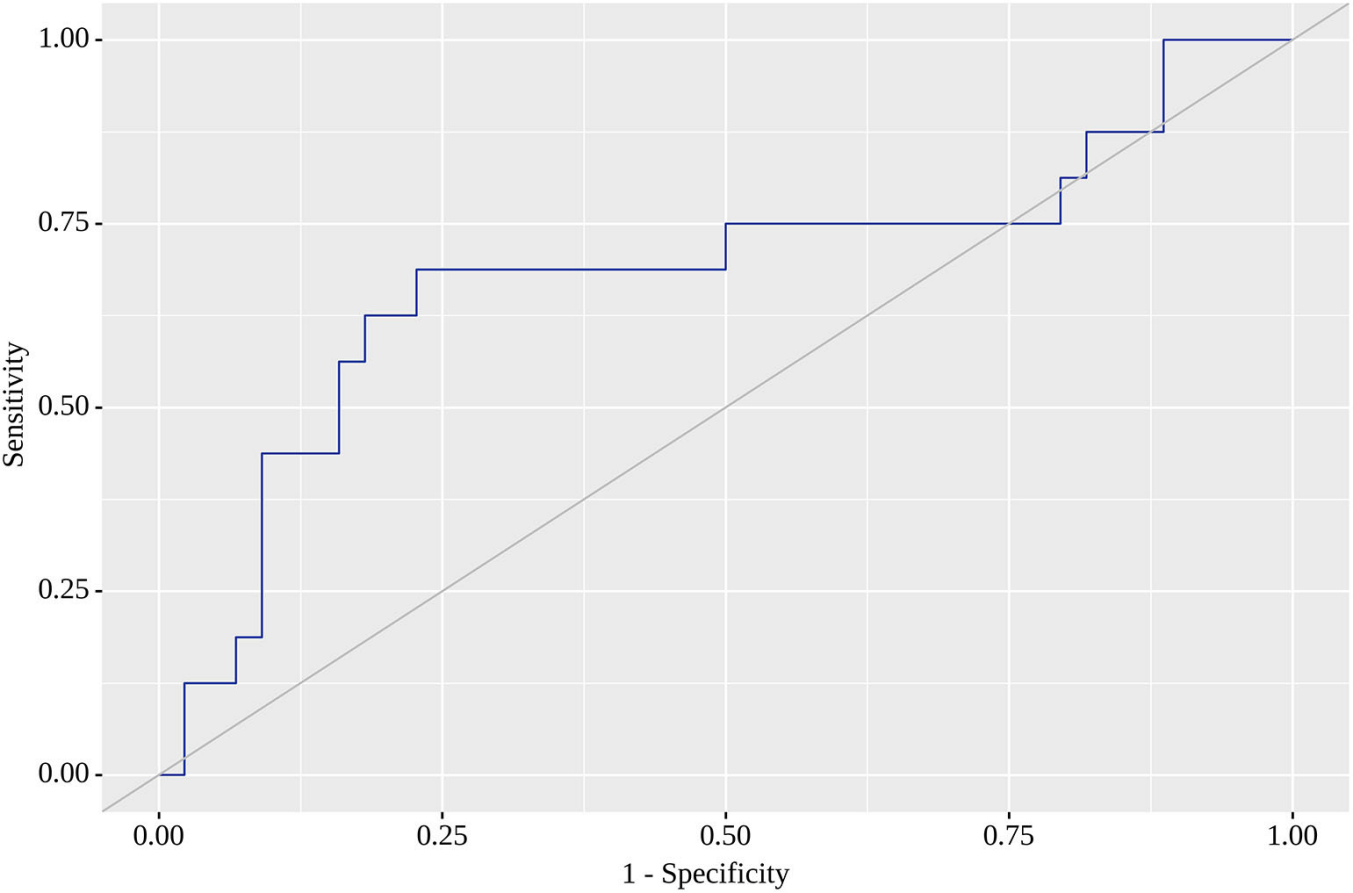
Receiver operating characteristic (ROC) curve characterizing positive outcome in right lumbar region (RRL).

**Figure 3.**
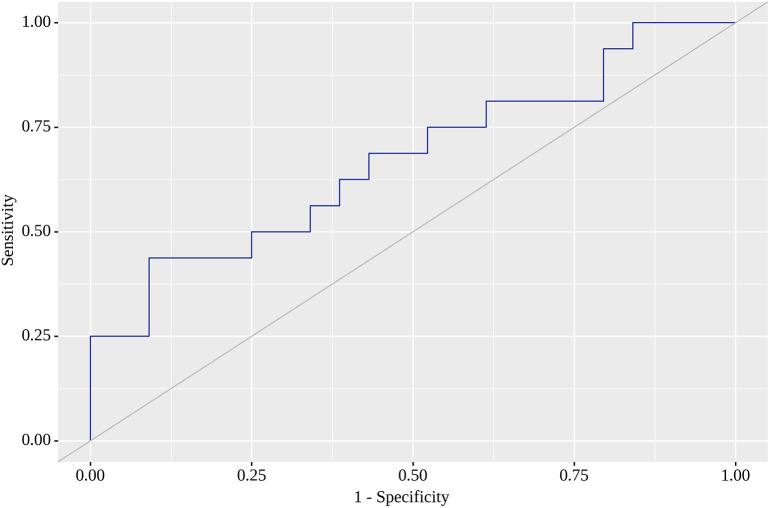
Receiver operating characteristic (ROC) curve showing positive outcome in umbilical region (RU).

**Figure 4.**
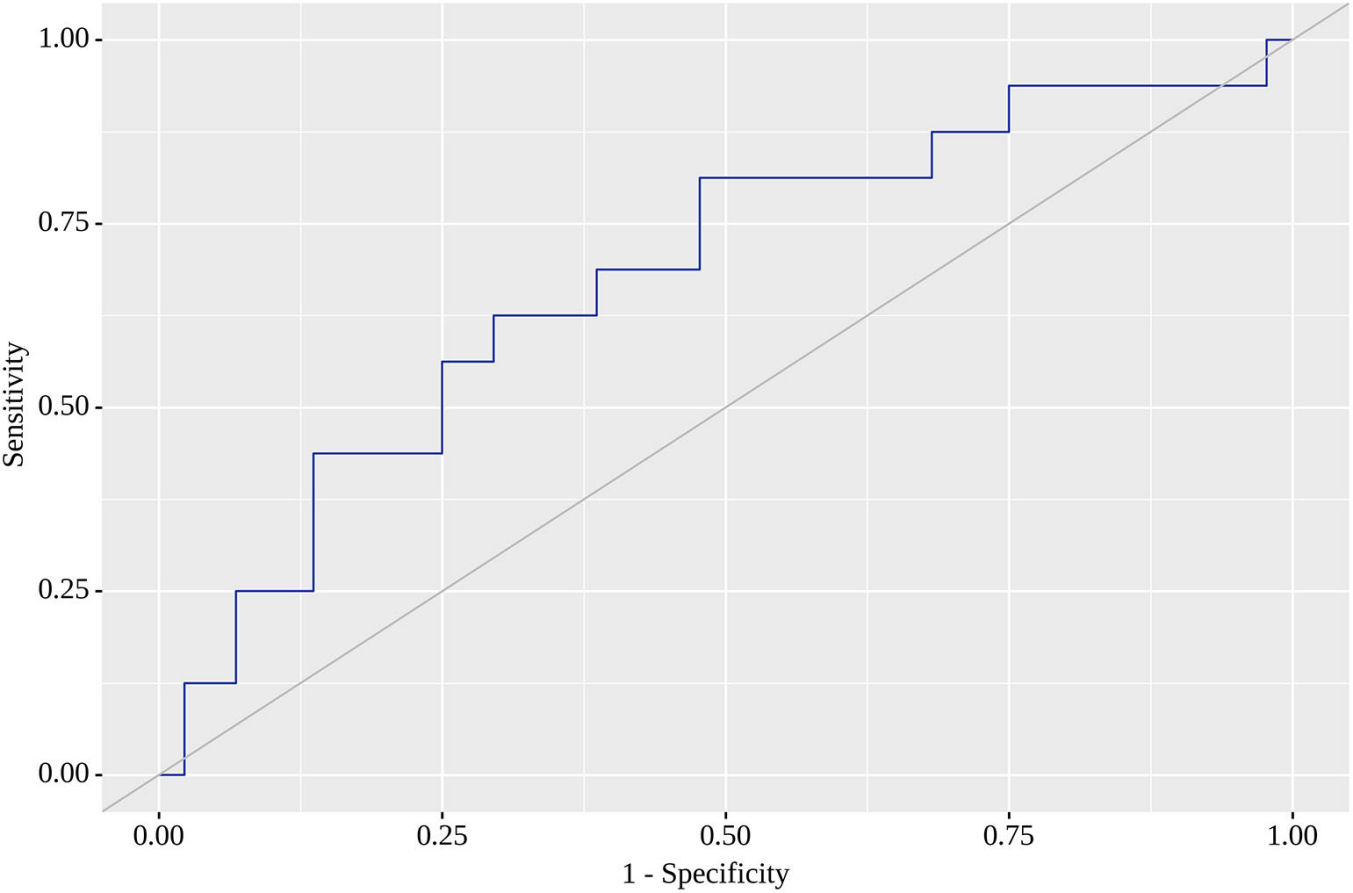
Receiver operating characteristic (ROC) curve characterizing positive outcome in right inguinal region (RRI).

Of the nine locations in which we tested SUV _max_ as a predictor of metastasis on the follow-up visit, the highest areas under the curve (AUC) were found for RLH, RRL, RU and RRI. For RLH, SUV _max_ of 0.74 yielded the greatest AUC (0.668; 95% CI 0.505 – 0.831) with quite high sensitivity (75%) and specificity (61%). Although this model was statistically significant (p=0.049) ( Figure 1), we failed to identify SUV _max_ corresponding to high sensitivity (80% or above) with acceptable specificity. When a high sensitivity of 80% was reached, we observed a dramatic fall in specificity. The corresponding SUV _max_ value with the highest AUC (0.682; 95% CI 0.520 – 0.843) for RRL was 1.05, for which sensitivity reached 69% and specificity was as high as 77%. This model was also statistically significant (p=0.032) ( Figure 2). SUV _max_ value with the highest AUC (0.672; 95% CI 0.509 – 0.835) for RU was 0.85, for which sensitivity equaled 63% with almost similar specificity (61%). This model was also statistically significant (p=0.043), and Figure 3 illustrates AUC for this location. Finally, SUV _max_ with the highest AUC (0.679; 95% CI 0.517 – 0.841) for RRI was 0.78, for which sensitivity reached 69%, but specificity was only 61%, but statistically significant (p=0.035). Figure 4 reflects AUC for this analysis. Finally, PTL, tumor stage system, tumor grade and staging on LM did not affect SUV _max_.

## Discussion

The current prospective observational cohort study is one of very few to identify the location of greater ^18^F-FDG accumulation by functional VAT activity as early markers of later metastases indicative of the metastatic status of CRC patients. In a cohort of 60 patients in adjusted regression models and ROC analysis we showed that ^18^F-FDG accumulation in RLH, RU and RRL and RRI were predictors of later metastases in CRC patients with moderate, but statistically significant sensitivity and specificity values. The threshold value of SUV _max_ 0.74 for RLH resulted in 75% sensitivity and 61% specificity, whereas the corresponding SUV _max_ for RRI was 0.78 with a sensitivity of 69% and a specificity of 61%. We also found that a threshold value of SUV _max_ 1.050 resulted in 69% sensitivity and 77% specificity for accumulation in RRL, whereas the SUV _max_ value of 0.85 warranted 63% sensitivity and 61% specificity for RU. In our analysis, ^18^F-FDG accumulation in the remaining tested five locations was not associated with later metastases risk.

The predictive value of ^18^F-FDG PET/CT for CRC has been reported in a number of preceding studies, reporting different SUV _max_ values. Byung Wook Choi *et al.* retrospectively emphasized the predictive value of metabolic parameters on ^18^F-FDG PET/CT in classical rectal adenocarcinoma in 149 patients on two models (AUC 0.778 and 0.762, p=0.04; 0.814 and 0.779, p=0.83). ^12^ One more study by Sung Hoon Kim *et al.* retrospectively showed the prognostic value of ^18^F-FDG PET/CT for LN metastasis in rectal cancer in 166 patients, nodal SUV _max_ 2.356, AUC 0.698 (p=0.04), 0.720 (0.033), 0.806 (p=0.04). ^18^ Finally, Kisoo Pahk *et al.* retrospectively showed the prognostic role of functional VAT activity evaluated by preoperative ^18^F-FDG PET/CT for regional or distant LN metastasis in 131 CRC patients; however, the ratio of visceral to subcutaneous fat activity (VAT/SAT) was evaluated, while the ratio of SUV _max_ 1.88, AUC 0.862, sensitivity 84.6%, specificity 78.8%, p<0.001. ^1^ Emir Sokolović *et al.* showed the predictive metabolic value of SUV _max_ with metastatic CRC patients, and concluded that SUV _max_ could be used as a novel predictive factor of disease progression among metastatic CRC patients. Average ±SD progression-free survival with a SUV _max_ above 4.1 was 11.3±9.37 months, and a SUV _max_ below 4.1 was 19.6±12.05 months (p=0.001). ^22^ Esra Arslan *et al.* showed the predictive potential of ^18^F-FDG PET/CT and KRAS mutation in CRC, where the mean SUV _max_ with primary tumor was estimated to be 21.1±9.1 (range= 6.0–47.5) and tumor mean SUV _max_ with a KRAS mutation (24.0±9.0) was found to be significantly higher than those without a KRAS mutation (17.7±8.2) (p=0.001). ^23^

A number of prior reports ascertained the relationship between visceral adiposity and the prediction of CRC. ^24^ Nevertheless, the outcomes were versatile and did not reach consent. These analyses used CT to measure VAT volume as a surrogate marker of VAT activity. But, VAT volume is reportedly unrelated to the visceral fat inflammatory process, ^25^ whereas the identification of VAT volume by CT may not be satisfactory in affecting the current functional VAT activity. ^5^ Therefore, a functional imaging modality like ^18^F-FDG PET/CT could be more suitable for evaluation of functional VAT activity than CT.

Previous research on functional VAT activity and ^18^F-FDG PET/CT concentrated on systemic inflammatory diseases, such as atherosclerosis or chronic obstructive pulmonary disease. ^5^ Liang-qian Tong *et al.* illustrated the association between pulmonary ^18^F-FDG metabolism and smoking history in 347 healthy adults with chronic obstructive pulmonary disease where differences in the pulmonary SUV _max_ according to smoking status were analyzed. The mean SUV _max_ of current smokers was higher than that of ex-smokers with a medium (1.03±0.14 vs 0.88±0.16) or larger tobacco burden (1.08±0.15 vs 0.89±0.11) (p=0.012, p<0.001, respectively). However, there were no differences between the mean SUV _max_ of ex-smokers (0.91±0.13) and current smokers (0.91±0.16) with a smaller tobacco burden (p=0.888). The mean SUV _max_ of ex-smokers and current smokers with less tobacco burden were both significantly higher than that of non-smokers (0.78±0.13) (p<0.001, p<0.001, respectively). ^26^

This research used ^18^F-FDG PET/CT to demonstrate the practical application of functional VAT activity for cancer disease, which can provide molecular data about inflammatory processes in CRC LM.

The current analysis has some limitations. Firstly, despite its prospective design, the study sample was limited, although patients were consecutively recruited for several years. Secondly, we could only enroll patients from a single nuclear medicine unit and only in the country’s capital. However, PET/CT is not yet widely available elsewhere in the country; therefore, the current sample is comprised of patients who were forced to travel to the capital for the examination, thus, representing a population from almost the entire country. Thirdly, predictive value was evaluated for SUV _max_ only, and other crucial factors such as grade, and location of the primary tumor could not be analyzed. Further prospective research data with larger populations will be necessary to verify our outcomes.

Finally, functional VAT activity evaluated by ^18^F-FDG PET/CT is substantially associated with LM. Furthermore, it is a useful factor for the prediction of LM. Implementing the results into practical medicine will help practitioners choose tactics and control CRC patients.

## Consent

All patients provided written informed consent for the study and participation.

## Data availability

### Underlying data

Open Science Framework: ‘Raw Underlying Data Colorectal Cancer for Predictive Value’. https://doi.org/10.17605/OSF.IO/NSZFK. ^21^

This project contains the following underlying data:

• Raw Underlying Data CRC for PV.xlsx (Demographic and preparation accumulation data in the studied cohort)

### Reporting guidelines

Open Science Framework: TREND checklist for ‘Predictive value CRC’ https://doi.org/10.17605/OSF.IO/PRM95. ^19^

Data are available under the terms of the Creative Commons Zero “No rights reserved” data waiver (CC0 1.0 Public domain dedication).

## References

[1] Pahk K Rhee S Kim S : Predictive Role of Functional Visceral Fat Activity Assessed by Preoperative F-18 FDG PET/CT for Regional Lymph Node or Distant Metastasis in Patients with Colorectal Cancer. *PLoS One.* 2016 Feb 10;11(2):e0148776. 10.1371/journal.pone.0148776 26862754 PMC4749214

[2] Xi Y Xu P : Global colorectal cancer burden in 2020 and projections to 2040. *Transl. Oncol.* 2021 Jul 6;14(10):101174. 10.1016/j.tranon.2021.101174 34243011 PMC8273208

[3] Bray F Ferlay J Soerjomataram I : Global cancer statistics 2018: GLOBOCAN estimates of incidence and mortality worldwide for 36 cancers in 185 countries. *CA Cancer J. Clin.* 2018 Nov;68(6):394–424. 10.3322/caac.21492 30207593

[4] Sung H Ferlay J Siegel RL : Global Cancer Statistics 2020: GLOBOCAN Estimates of Incidence and Mortality Worldwide for 36 Cancers in 185 Countries. *CA Cancer J. Clin.* 2021;71(3):209–249. 10.3322/caac.21660 33538338

[5] Bucerius J Vijgen GHEJ Brans B : Impact of Bariatric Surgery on Carotid Artery Inflammation and the Metabolic Activity in Different Adipose Tissues. *Medicine (Baltimore).* 2015 May 22;94(20):e725. 10.1097/MD.0000000000000725 25997038 PMC4602867

[6] Ozis SE Soydal C Akyol C : The role of 18F-fluorodeoxyglucose positron emission tomography/computed tomography in the primary staging of rectal cancer. *World J. Surg. Oncol.* 2014 Feb 1;12(1):26. 10.1186/1477-7819-12-26 24484935 PMC3912933

[7] Suzuki Y Okabayashi K Hasegawa H : Metabolic Tumor Volume and Total Lesion Glycolysis in PET/CT Correlate With the Pathological Findings of Colorectal Cancer and Allow Its Accurate Staging. *Clin. Nucl. Med.* 2016 Oct;41(10):761–765. 10.1097/RLU.0000000000001332 27556789

[8] Hong JH Kim HH Han EJ : Total Lesion Glycolysis Using 18F-FDG PET/CT as a Prognostic Factor for Locally Advanced Esophageal Cancer. *J. Korean Med. Sci.* 2016 Jan;31(1):39–46. 10.3346/jkms.2016.31.1.39 26770036 PMC4712578

[9] Bang JI Ha S Kang SB : Prediction of neoadjuvant radiation chemotherapy response and survival using pretreatment [18F] FDG PET/CT scans in locally advanced rectal cancer. *Eur. J. Nucl. Med. Mol. Imaging.* 2016 Mar 1;43(3):422–431. 26338180 10.1007/s00259-015-3180-9

[10] Ogawa S Itabashi M Kondo C : Prognostic Value of Total Lesion Glycolysis Measured by 18F-FDG-PET/CT in Patients with Colorectal Cancer. *Anticancer Res.* 2015 Jun;35(6):3495–3500. 26026116

[11] Shi D Cai G Peng J : The preoperative SUVmax for 18F-FDG uptake predicts survival in patients with colorectal cancer. *BMC Cancer.* 2015 Dec 21;15(1):991. 26689966 10.1186/s12885-015-1991-5 PMC4687154

[12] Choi BW Kang S Bae SU : Prognostic value of metabolic parameters on 18F-fluorodeoxyglucose positron tomography/computed tomography in classical rectal adenocarcinoma. *Sci. Rep.* 2021 Jun 21;11(1):12947. 10.1038/s41598-021-92118-x 34155222 PMC8217562

[13] Deantonio L Caroli A Puta E : Does baseline [18F] FDG-PET/CT correlate with tumor staging, response after neoadjuvant chemoradiotherapy, and prognosis in patients with rectal cancer? *Radiat. Oncol.* 2018 Oct 25;13(1):211. 10.1186/s13014-018-1154-3 30359275 PMC6202838

[14] Park JS Huh JW Park YA : Prognostic Comparison Between Mucinous and Nonmucinous Adenocarcinoma in Colorectal Cancer. *Medicine (Baltimore).* 2015 Apr 17;94(15):e658. 10.1097/MD.0000000000000658 25881840 PMC4602499

[15] Boellaard R Delgado-Bolton R Oyen WJG : FDG PET/CT: EANM procedure guidelines for tumour imaging: version 2.0. *Eur. J. Nucl. Med. Mol. Imaging.* 2015;42(2):328–354. 10.1007/s00259-014-2961-x 25452219 PMC4315529

[16] Milardovic R Beslic N Sadija A : Role of 18F-FDG PET/CT in the Follow-up of Colorectal Cancer. *Acta Inform. Medica.* 2020 Jun;28(2):119–123. 10.5455/aim.2020.28.119-123 PMC7382771 32742064

[17] Niccoli Asabella A Simone M Ballini A : Predictive value of 18F-FDG PET/CT on survival in locally advanced rectal cancer after neoadjuvant chemoradiation. *Eur. Rev. Med. Pharmacol. Sci.* 2018 Dec;22(23):8227–8236. 10.26355/eurrev_201812_16517 30556862

[18] Kim SH Song BI Kim BW : Predictive Value of [18F] FDG PET/CT for Lymph Node Metastasis in Rectal Cancer. *Sci. Rep.* 2019 Mar 21;9:4979. 10.1038/s41598-019-41422-8 30899056 PMC6428820

[19] Suleimanov A Saduakassova A Vinnikov D : TREND Checklist - Predictive Value CRC. 2022 Aug 1 [cited 2022 Sep 15]. Reference Source

[20] Boellaard R Delgado-Bolton R Oyen WJG : FDG PET/CT: EANM procedure guidelines for tumour imaging: version 2.0. *Eur. J. Nucl. Med. Mol. Imaging.* 2015 Feb;42(2):328–354. 10.1007/s00259-014-2961-x 25452219 PMC4315529

[21] Suleimanov A Saduakassova A Vinnikov D : Raw Underlying Data Colorectal Cancer for Predictive Value. 2022 Jul 22 [cited 2022 Sep 15]. Reference Source

[22] Sokolović E Cerić T Cerić Š : The Prognostic Value of SUVmax of 18F-FDG PET/CT in Patients with Metastatic Colorectal Cancer. *Acta Medica Acad.* 2020 Apr;49(1):1–8. 10.5644/ama2006-124.278 32738112

[23] Arslan E Aksoy T Gürsu RU : The Prognostic Value of 18F-FDG PET/CT and KRAS Mutation in Colorectal Cancers. *Mol. Imaging Radionucl. Ther.* 2020 Feb;29(1):17–24. 10.4274/mirt.galenos.2019.33866 32079384 PMC7057728

[24] Rickles AS Iannuzzi JC Mironov O : Visceral Obesity and Colorectal Cancer: Are We Missing the Boat with BMI? *J. Gastrointest. Surg.* 2013 Jan 1;17(1):133–143. 10.1007/s11605-012-2045-9 23090279

[25] Christen T Sheikine Y Rocha VZ : Increased glucose uptake in visceral versus subcutaneous adipose tissue revealed by PET imaging. *JACC Cardiovasc. Imaging.* 2010 Aug;3(8):843–851. 10.1016/j.jcmg.2010.06.004 20705265 PMC4042675

[26] Qian TL Fang SY Nan JS : The Association Between Lung Fluorodeoxyglucose Metabolism and Smoking History in 347 Healthy Adults. *J. Asthma Allergy.* 2021 Mar 31;14:301–308. 10.2147/JAA.S302602 33840997 PMC8032449

