# Supplementary material for: Predictive value of 18F-fluorodeoxyglucose accumulation in visceral fat activity to detect colorectal cancer metastases (prospective observational cohort study)
Source: F1000Res. 2022 Oct 10;11:1158. [Version 1] doi: 10.12688/f1000research.122814.1 (PMC10285332; doi:10.12688/f1000research.122814.1)
Supplement: Peer review history

# Peer review: Reviewer response for version 1

**DOI:** 10.5256/f1000research.134853.r175503

**Published:** 2023-06-13

**Authors:** Marianie Musarudin

**Affiliations:** 1 SCHOOL OF HEALTH SCIENCES, Universiti Sains Malaysia, Health Campus, Kelantan, Malaysia

This study highlights the potential for early LN metastases detection, by quantitatively measures the functional VAT activity using ^18^F-FDG PET/CT.  Eight subdomains of abdominal regions and pelvic cavity was included in the assessment. The study utilised ROC analysis as the statistical method.

 Some clarifications needed:

1. "The average activity dose of the injected 18F-FDG was 252.55 **MBk**, ranging from 132.5 to 465.3 **MBk**." ----> Use SI unit, MBq.
2. The VAT areas were identified by using predefined HU, ranging from -70 to -110. Justify this selection with citation.
3. You have calculated the sample size. Is the sample size of 60 actually less than the estimated sample number? If yes, according to the sample size calculation, how much sample is actually needed? Some authors use a flow chart to show the study design and present the sample size and excluded sample number (with the causes). 
4. Some studies considered total adipose tissue SUV (SUV _TAT_) or relative SUV (SUV _VAT_ normalized to the SUV _TAT_) for their analysis. Add a sentence explaining why SUV _MAX_ was used in this study. 

Is the work clearly and accurately presented and does it cite the current literature?

Yes

If applicable, is the statistical analysis and its interpretation appropriate?

I cannot comment. A qualified statistician is required.

Are all the source data underlying the results available to ensure full reproducibility?

Yes

Is the study design appropriate and is the work technically sound?

Yes

Are the conclusions drawn adequately supported by the results?

Yes

Are sufficient details of methods and analysis provided to allow replication by others?

Yes

Reviewer Expertise:

Nuclear Medicine

I confirm that I have read this submission and believe that I have an appropriate level of expertise to confirm that it is of an acceptable scientific standard.

# Peer review: Reviewer response for version 1

**DOI:** 10.5256/f1000research.134853.r156188

**Published:** 2022-12-08

**Authors:** Jandos Amankulov

**Affiliations:** 1 Department of Radiology and Nuclear Medicine, Kazakh Institute of Oncology and Radiology, Almaty, Kazakhstan

This article addresses the relationship between 18F-fluorodeoxyglucose uptake level in visceral adipose tissue (VAT) and the development of metastasis in colorectal cancer (CRC) patients. It addresses an important topic as early predictor markers of metastasis can improve the prognosis of the disease. Despite the rather small sample size noted by the authors, this study will be the beginning of further research on large samples.

 However, there are a number of issues that need to be clarified/corrected:

1. Please use MBq as the unit of measurement instead of MBk.
2. Please use mmol/L as the unit of measurement instead of mmol/l, as a broader group more easily recognizes the capitalized version.
3. Many studies have extensively analyzed the effect of blood glucose levels and other confounding factors on 18F-FDG uptake in normal tissue and tumor. In your research, have you corrected the VAT SUVmax measurements based on the participant’s blood glucose levels in normoglycemia?
4. The study results do not support the statement that implementation of VAT 18F-FDG uptake measurement will improve CRC patients’ management. The clinical utility/usefulness of this biomarker was not assessed within this study. This hypothesis would need to be validated in other non-inferiority trials. So use “would/might help” instead of " *will help*" in the sentence " *Implementing the results into practical medicine will help practitioners choose tactics and control CRC patients*",  or remove this sentence.

Is the work clearly and accurately presented and does it cite the current literature?

Yes

If applicable, is the statistical analysis and its interpretation appropriate?

Yes

Are all the source data underlying the results available to ensure full reproducibility?

Yes

Is the study design appropriate and is the work technically sound?

Yes

Are the conclusions drawn adequately supported by the results?

Yes

Are sufficient details of methods and analysis provided to allow replication by others?

Yes

Reviewer Expertise:

Radiology and Nuclear Medicine in Cancer Diagnosis

I confirm that I have read this submission and believe that I have an appropriate level of expertise to confirm that it is of an acceptable scientific standard.
